# Historical Perspectives and Evolution of Menstrual Terminology

**DOI:** 10.3389/frph.2022.820029

**Published:** 2022-02-28

**Authors:** Rohan R. Chodankar, Malcolm G. Munro, Hilary O. D. Critchley

**Affiliations:** ^1^Department of Obstetrics and Gynaecology, NHS Lothian, Edinburgh, United Kingdom; ^2^Department of Obstetrics and Gynecology, David Geffen School of Medicine at UCLA, Los Angeles, CA, United States; ^3^Medical Research Council Centre for Reproductive Health, The Queen's Medical Research Institute, The University of Edinburgh, Edinburgh, United Kingdom

**Keywords:** menstruation, menstrual terminology, abnormal uterine bleeding, PALM-COEIN, menstrual disorders

## Abstract

Abnormal uterine bleeding (AUB) in the reproductive years in non-pregnant women comprises a group of symptoms that include abnormal frequency and the irregular onset of flow as well as prolonged and heavy menstrual bleeding. It is a common, chronic, and debilitating condition affecting women worldwide with an adverse impact on their quality of life. Until the last decade, the “menstrual” terminology used to describe both normal and abnormal uterine bleeding and its underlying causes was inconsistent, creating considerable confusion. Using standardized terminology may potentially improve clinical management as well as help designing and interpreting basic, translational, epidemiological, and clinical research in women with menstrual problems. In this article, we explore the history and evolution of menstrual terminology and discuss the two International Federation of Gynecology and Obstetrics (FIGO) systems on i.e., (A) menstrual terminology and definitions (B) and the causes of AUB, achieved through international consensus of relevant stakeholders through a long multistage journey.

## Introduction

Abnormal uterine bleeding (AUB) in the reproductive years in non-pregnant women comprises a group of symptoms that include abnormal frequency and the irregular onset of flow as well as prolonged and heavy menstrual bleeding; the latter referred to as HMB. Individually or collectively, the symptoms frequently have an adverse effect on the quality of life (QoL) and can be debilitating. The precise prevalence of AUB is not well-understood since many women normalize their symptoms, do not present for care, or are deemed “normal” by healthcare providers, but it has been estimated that at least 1 in 4 women of reproductive age are affected, however the prevalence may be as high as 53% (1–3). It is important to remember that AUB is a collection of symptoms and that, in each instance, there exists one or more underlying causes that are almost always benign, but occasionally, and especially in the later reproductive years, may be premalignant or malignant. Heavy menstrual bleeding especially is typically chronic, and in addition to the cyclical adverse impact on QoL, the chronic blood loss frequently leads to iron deficiency with all the attending adverse effects on cognitive and physical function (4).

Until the last decade, the “menstrual” terminology used to describe both normal and abnormal uterine bleeding and its underlying causes was inconsistent, leading to the widespread use of a variety of poorly defined terms. In the past, this circumstance hampered both teaching and clinical management and made challenging the process of designing and interpreting basic, translational, epidemiological, and clinical research in women with menstrual disorders. A well-known example includes two contemporaneous clinical trials, in the USA and in Europe, established to answer the same clinical question (5) due to lack of clarity on menstrual disorder terminology.

In this article, we explore the history of menstrual terminology, the potential causes of AUB symptoms, the continuing evolution to the current versions of the two systems developed by the International Federation of Gynecology and Obstetrics (FIGO) as per the FIGO Committee on Menstrual Disorders, known as the MDC.

## Historical Perspectives

Although it is beyond the scope of this chapter to explore all the historical perspectives associated with abnormal menstruation, we discuss below the presumed origin of three of the terms commonly used in the medical literature to describe menstrual disorders, i.e., menorrhagia, metrorrhagia, and dysfunctional uterine bleeding. It is difficult to ascribe these above-mentioned terms to the exact descriptions in the historical texts as discussed below. Much of this history of menstrual terminology is addressed in depth in the publication by Woolcock et al. (6).

In the early literature (430BC until the 1800s), what is currently defined as HMB was described variously as “excessive evacuations of the menses, inordinate flowing, the immoderate flux, an overflowing of the courses, excessive flooding's, uterine hemorrhage, and so on.” Hippocrates (born around 460 BC) in his Aphorisms, translated from Greek and Latin to English in 1822 (potentially addresses HMB in the following descriptions: “To stop excessive evacuations of the menses, a large cupping glass may be applied to the breast,” and “Menstruation if too abundant produces disease” (7).

The popular Greek philosopher Aristotle (Third century BC) also addressed excessive menstrual bleeding, as referenced in the English translations of his work Aristotle's Masterpieces, although it is believed that he relied heavily upon the works of Hippocrates for medical reference. For example: “In quantity, bleeding is excessive, saith Hippocrates, when they flow about eighteen ounces;” “In time when they flow about 3 days;” and “but it is inordinate flowing when the faculties of the body are thereby weakened.” These menstrual volumes fit with those of women in clinical trials of drugs and devices designed to treat causes of HMB, and the “weakened faculties” could be perceived to be the result of iron deficiency!

Other historical references include the Bible (New Testament, Gospel of St. Mark, King James I translation from the original Greek, 1611) where excessive bleeding is described as “And a woman, which had an issue of blood 12 years, and had suffered many things of many physicians and straightway the fountain of her blood was dried up.”

Avicenna, the Persian philosopher, via his book Canon of Medicine describes a scenario where “menstruation is profuse and is arrested with difficulty.” In 1666, Thomas Sydenham, the English physician, when addressing “immoderate menstrual flow” described how “the natural flow of the menses would fill a vessel the size of a goose's egg,” perhaps reflecting a desire to communicate the quantity of blood lost at menstruation. Furthermore, the same author describes that “when inordinate, there is difficulty, weakness, anorexia, cachexia, cadaverous complexion, and swelling of the feet.” The latter content may be capturing the symptoms of (gross) anemia associated with heavy menstrual loss. None of the historical publications concerning menstruation used the term “heavy menstrual bleeding” or “menorrhagia,” but clearly addressed the symptom through other descriptors (6).

The term “menorrhagia” is believed to have been first used by Professor William Cullen, Professor of the Practice of Physic at the University of Edinburgh, in the 1700s. Its usage appears in his textbook of lectures to medical students (8). One of the earliest written uses of the term was in a discourse in Latin written by one of his student's and attributed to Cullen. The word “menorrhagia” is derived from the Greek noun “mene” meaning moon, and the verb “regnumi” meaning to burst forth, to let loose or break asunder, the implication being sudden severe blood loss. Cullen also used the term “maetrorrhagia” in his lectures. The origin is from the Greek noun, “metra,” meaning uterus, and the verb “regnumi” again, perhaps suggesting bleeding bursting forth from the uterus at any time, that is, much less regular than implied by “menorrhagia.” The English physician Fleetwood Churchill (one of the first true specialist obstetrician/gynecologists clearly summarizes early nineteen century use of the term “menorrhagia” in his textbook on “Principal Diseases of Females. There in, “metrorrhagia” appears to have been a less popular term than “menorrhagia,” and Churchill omits use the term (6).

The causes of menstrual disorders receive considerably less attention in historical literature before the 1800s, predominantly attributed to the lack of knowledge. During the late nineteenth century and early twentieth century that the causes of AUB were starting to be recognized. With the advent of anesthetic safety, histological assessments and radiology, the causes of AUB were becoming more apparent. The confusing term “dysfunctional uterine bleeding,” or DUB, did not appear until the 1930s. it is then that possible causes for AUB in a group of women who did not have recognizable local pelvic pathology began to be considered.

## The Problem With Traditional Menstrual Terminology

There is considerable confusion in the existing medical literature when describing normal menstrual bleeding and AUB symptoms and distinguishing those symptoms from their underlying etiology. In the past, and too often in the present, terms such as HMB and AUB, and previously, menorrhagia and DUB, have been often used to indicate either or both a symptom and a diagnosis. Such a circumstance can adversely impact the design and interpretation of clinical and basic research, and, thereby, undermine clinical care. Historically, the two most common descriptors used are the terms menorrhagia and DUB, and we will use these as examples to highlight the problem with menstrual terminology that ultimately led to the design of the two FIGO systems.

The term “menorrhagia” appears to have been universally employed as a description of some aspect of excessive, heavy, or prolonged menstrual blood loss; however, no clear definition existed. Woolcock et al. (6) reviewed 100 articles (in English) appearing on Medline (Ovid Technologies, Inc, New York, USA) between 2000 and 2006 where the term “menorrhagia” appeared in the article title. The articles were classified based on the usage of the term menorrhagia in 4 major categories:

- If the term menorrhagia was defined or not,
- If the term menorrhagia was used as a symptom of heavy uterine bleeding, with or without pathology, with irregular or regular bleeding,
- If the term menorrhagia was recognized as a patient complaint or a doctors' definition,
- If the term menorrhagia was used as a diagnosis by itself or in combination with other adjectives (see Table 1).

**Table 1:** Analysis of the use of the term menorrhagia.

**Category**	**Usage**
1(a) Defined	56
1(b) Undefined	44
	*n* = 100
2(a) Used as symptom of heavy uterine bleeding, irregular or regular, with or without pathology	34
2(b) Used as symptom of heavy uterine bleeding, regular, with or without pathology	28
2(c) Used as a symptom of heavy uterine bleeding, regular with no detectable pathology	16
	*n* = 78
3(a) Primarily reflecting patient complaint	59
3(b) Primarily reflecting the doctor's definition	19
	*n* = 78
4(a) Used as a diagnosis	5
4(b) Used as a diagnosis when combined with another term (e.g., “idiopathic”)	17
	*n* = 22

*Adapted from Woolcock et al. (6)*.

The analysis of these 100 articles suggested that nearly 1 in 5 authors used the term menorrhagia to describe a diagnosis rather than a symptom and nearly 75% of these authors used a qualifying adjective preceding the term menorrhagia e.g., idiopathic, essential, and so on. Overall, the authors concluded that the use of the term was sometimes so uncertain that approximations had to be made as to which of the 4 categories was suitable with an overlap in several instances.

Similarly the term DUB in the UK referred to regular, (i.e., cyclic and predictable) HMB following the exclusion of other pathologies i.e., likely describing ovulatory bleeding. In the USA the term DUB usually referred to irregular uterine bleeding related to anovulation (9). The term DUB was first used by Graves in the 1930s to ascribe the “impairment of endocrine factors that normally control menstruation.” Whereas, the confusion in terminology is apparent, this lack of clarity may also impact the interpretation and implementation of clinical trial data. An example includes a UK-based randomized controlled trial RCT (*n* = 204) which randomized women with a clinical diagnosis of DUB to a hysterectomy or hysteroscopic surgery (endometrial resection or endometrial laser ablation). The final histology however, revealed the presence of fibroids, adenomyosis and endometrial cancer, a circumstance that reflects the diagnostic heterogeneity of the enrolled subjects (10). The inclusion of such intervention based RCTs in systematic reviews, and, if performed, meta-analysis, can produce misleading results, as the primary inclusion criteria could be considered flawed since they were based on a symptom such as DUB rather than the underlying cause of the symptom.

## Evolution Of Menstrual Terminology

Achieving an international consensus on menstrual terminology has been a multistage journey. The process was designed to include a wide spectrum of stakeholders representing national and subspecialty gynecological societies worldwide, relevant medical journals, the FDA, and a variety of recognized experts from six continents. The initial result was a consensus-based system that defined both normal and abnormal menstrual bleeding with simple terms translatable into multiple languages. Ultimately, the process evolved to include a second system classifying the potential causes or contributors to AUB symptoms and called the PALM-COEIN system. In 2011 the two systems were initially presented together in a seminal paper that was then updated in 2018 following an additional rigorous process of clarification and revision (11, 12). The entire process was initially conducted under the aegis of a FIGO Menstrual Disorders Working Group, that subsequently became the Committee on Menstrual Disorders (usually called the Menstrual Disorders Committee or MDC).

- Terminology and Definitions (FIGO-AUB System 1)
- Classification of Causes of AUB in the Reproductive Years, the PALM-COEIN system (FIGO-AUB System 2).

**Figure 1:**
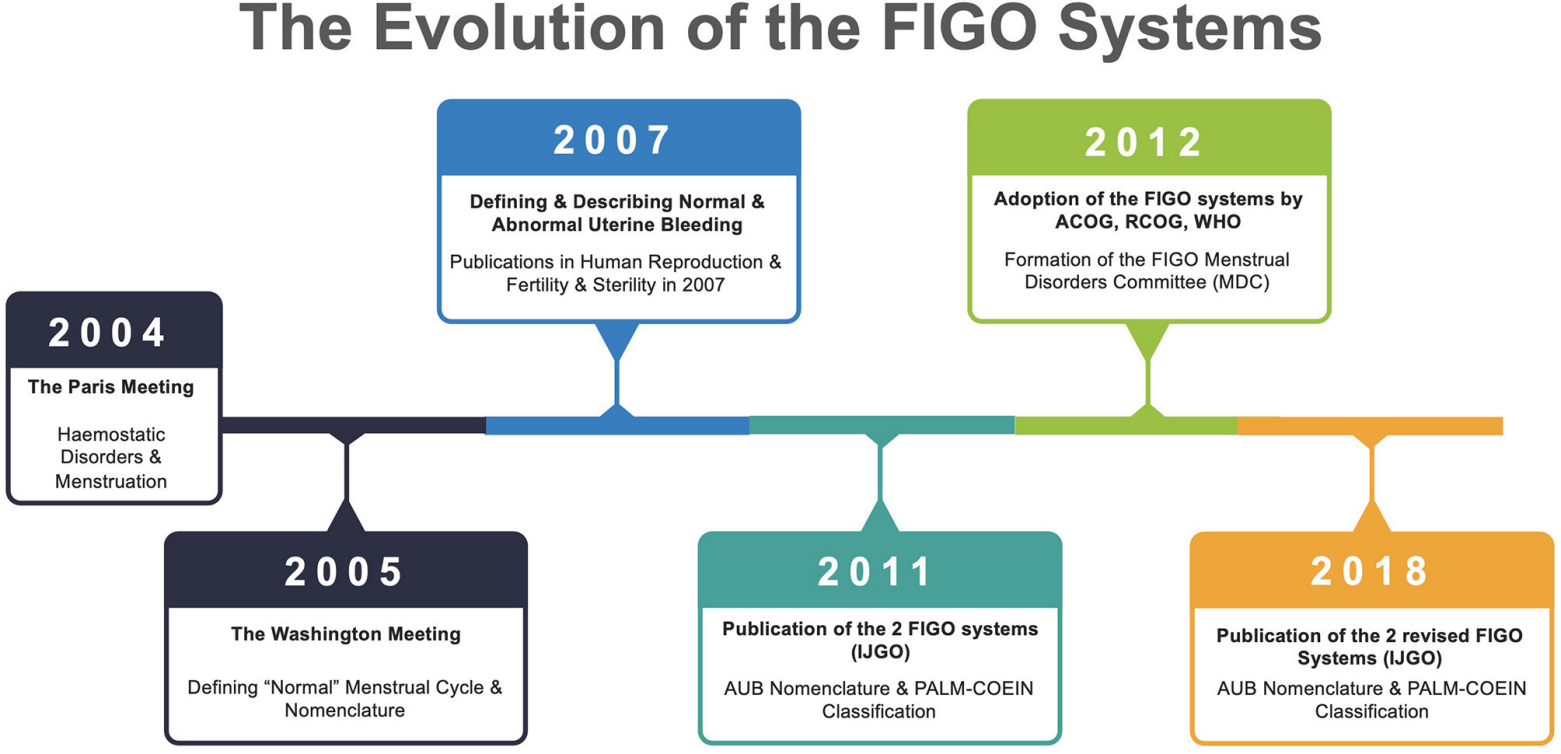
Evolution of the two FIGO Systems. Relevant publications include 2005 (13–15), 2007 (16, 17), 2008 (6), 2011 (11), and 2018 (12). FIGO, International Federation of Gynecology and Obstetrics; IJGO, International Journal of Gynecology & Obstetrics; ACOG, American College of Obstetricians & Gynecologists; RCOG, Royal College of Obstetricians & Gynecologists; WHO, World Health Organization; AUB, Abnormal Uterine Bleeding; MDC, Menstrual Disorders Committee.

The evolution of this process is shown in Figure 1.

### The Paris Meeting

The first step in the development of a standardized system was to deal with AUB associated with systemic disorders of hemostasis. The core group began by assembling an international group of clinician-investigators from the gynecological and hematological communities with expertise in the field of AUB and/or inherited haemostatic disorders. The goals developed for the group were:

1. Collaborative review of the evidence base concerning the prevalence and clinical impact of disorders of haemostasis in reproductive-aged females with AUB.
2. Development of a consensus on an appropriate screening methodology and tests of coagulation function suitable for use in the evaluation of females with AUB.
3. Evidence-based evaluation of AUB therapeutic approaches in females with known disorders of haemostasis.
4. Identification and prioritization of targets for clinical and basic research in the future.

Following development of draft documents, the members of the interdisciplinary consensus group assembled in Paris, France in May 2004. It was a less formal process that started with presentations and was followed by group discussion. Recommendations required the consensus of members and areas of disagreement were recorded. Following the meeting, manuscripts were drafted and circulated to subgroup members for required revisions. Each manuscript was distributed to each member of the consensus group for approval. This then culminated into the development of several important publications (13–15).

### The Washington Meeting

In 2004 the core organizers of the Paris meeting started to develop a process where the aim was to recommend clear, simple terminologies and definitions that would have the potential for wide acceptance. The process was called “Terminologies, Definitions and Classifications of Abnormal Uterine Bleeding (AUB)” and the aim was to determine consensus to support clinical care, trainee education, and the future design and interpretation of basic, translational, clinical and epidemiologic research related to non-gestational abnormal uterine bleeding in the reproductive years (16, 18).

The process began by performing a detailed literature review for terms commonly used to describe menstrual disorders (i.e., menorrhagia, dysfunctional uterine bleeding, and abnormal uterine bleeding) with the search including a variety of publications such as clinical trials, review articles, and well-read popular gynaecologic textbooks. This review confirmed that there was significant inconsistency and resulting confusion regarding the terminology used to describe normal and abnormal menstruation. With this material, the organizers sought and received support from FIGO, the American Society for Reproductive Medicine (ASRM) and the European Society of Human Reproduction and Embryology (ESHRE) and received unconditional grants from several donors. With this support, the organizers established contact with relevant international and national organizations, journal editors, representatives of the US Food and Drug Administration (FDA), and experts including reproductive endocrinologists, gynecologists, and investigators to develop an expert panel. Ultimately this panel comprised 35 representatives that included a broad spectrum of stakeholders including those from both developed and developing countries.

The Washington process included experts in the use of the RAND corporation's Delphi process (M. Broder and the Partnership for Health Analytic Research, Beverly Hills, CA). The Delphi method is a validated nominal group process designed to determine consensus on a clearly defined issue using a series of anonymous polls with individualized feedback designed to provide context in a non-confrontational fashion (19). For the Washington meeting a modification of the model was used that initially comprised a series of e-mail-based surveys of the panel members designed to determine their understanding of the use of terminology to describe normal and abnormal menstrual bleeding, as well as the causes of AUB in the reproductive years. The polls were designed so that most items were rated on a 4-point scale, and agreement was defined as at least 80% of respondents rating the item either 1 and 2, or 3 and 4. For example, if the rating scale was 1 = strongly disagree, 2 = disagree, 3 = agree, and 4 = strongly agree, at least 80% of respondents were required to provide either a “disagree” answer (1 or 2) or an “agree” answer (3 or 4) for there to be agreement on that item. Results were reported as the mean of the responses.

The aggregate ratings were shared when the expert group met in person for 3 days in February 2005 in Washington, D.C. (USA). Delphi rounds performed at the Washington meeting were conducted using an anonymous electronic survey system (Audience Response System) allowing for instantaneous polling of the participants and display of the results (11). The aggregate survey responses were considered in a plenary session of all meeting participants and also in smaller groups dedicated to aspects of classification and terminology.

Following extensive discussions, the smaller groups identified areas of agreement and disagreement, which were used to create new survey questions. These modified surveys were subsequently administered to all participants during a plenary session using electronic voting. During this In second round of ratings, two levels of agreement were identified. Panelists were considered to have “agreed” on an item if ratings met the original criteria (0.80% of answers were either 1 and 2 or 3 and 4). Panelists were considered to have “unanimously agreed” if all rated an item either 1 and 2 or 3 and 4 (e.g., 100% of respondents selected either 4, “strongly agree,” or 3, “agree”).

The “Washington” meeting and its Delphi process led to the following major outcomes:

- There was no consensus definition for terms such as menorrhagia, metrorrhagia, hypermenorrhea, and dysfunctional uterine bleeding.
- These terms and similar ones such as oligomenorrhea, polymenorrhea, hypermenorrhea, and others should be abandoned.
- Simple, descriptive terms with clear definitions should be used which should be understood by health professionals and patients alike, and importantly, any terminology adopted should be suitable for translation into most languages.
- These simple terms should describe the parameters of menstrual frequency, regularity, duration, and volume, with norms defined by the 5th to 95% centiles as determined by analyses of large menstrual databases (20, 21).
- There exists a need for a separate system designed to categorize the causes, not the symptoms, of non-gestational AUB in the reproductive years. General concepts and categories were discussed and debated and there was substantial support for a system that recognized structural causes as well as those that are secondary to non-structural disorders.

Following the Washington meeting the FIGO Menstrual Disorders Working Group (MDWG) was established in early 2006 and the results of the Delphi process published simultaneously in two journals, Fertility and Sterility and Human Reproduction, in 2007 (16, 17).

Based on the consensus developed in the Washington meeting, the MDWG recommended that the following terms (see Table 2) that have been used over the last 100 years or so should no longer be used (6, 11, 12, 16, 17).

**Table 2:** Terms used to describe menstrual disorders that should no longer be used.

•Anomalous uterine hemorrhage
•Anovulatory menorrhagia
•Dysfunctional uterine bleeding;
•Excessively heavy menstrual loss
•Epimenorrhea
•Epimenorrhagia
•Essential menorrhagia
•Functional uterine hemorrhage
•Functional menorrhagia
•Genuine menorrhagia
•Hypermenorrhea
•Idiopathic menorrhagia
•Idiopathic uterine hemorrhage
•Menorrhagia
•Meno-metrorrhagia
•Metropathia hemorrhagica
•Ovulatory menorrhagia
•Polymenorrhea
•Polymenorrhagia
•Primary menorrhagia
•Persistent menorrhagia
•Symptomatic menorrhagia
•Unexplained menorrhagia
•Uncomplicated menorrhagia

The MDWG had several activities relating to work surrounding menstrual terminology including presentations, publications, workshops, and meetings. However, and most importantly, it paved the way for planning a focused working group meeting during the 2009 FIGO World Congress in Cape Town and an AUB symposium was held within the main scientific program of the 2009 FIGO World Congress.

### The Cape Town Meeting

FIGO's 19th triennial World Congress of Gynecology and Obstetrics, held in October 2009 in Cape Town, South Africa, provided a further forum for a pre-congress menstrual disorders workshop. In preparation for the meeting the MDWG recruited additional participants and initiated development of a draft system for classification of potential causes supported by telephonic and person to person discussion. At the workshop, members of the MDWG discussed and refined the elements of the system for classification of causes of AUB in the reproductive years. Following that, in the main AUB symposium “Let us Talk about How We Can Improve Clinical Management through Clear Language and Disease Classification,” there was a unique opportunity to ascertain opinions concerning the proposed systems (definitions of symptoms, and classification of causes) from over 800 participants with diverse national and socioeconomic backgrounds, and aided by the use of an audience response system (ARS). This process was also designed to gauge the ability of participants from a spectrum of countries, including those defined as low and middle income countries (LMIC), to have the resources needed to evaluate patients using imaging and laboratory tests. The main outcomes from this meeting were as follows:

- 215/237 (90.7%) respondents agreed that “AUB” was a suitable overarching term for abnormal menstrual symptoms.
- 96/141 (68.1%) and 171/223 (76.7%), respectively, supported proposals that terms such as “menorrhagia” and “DUB” be discarded.
- 198/237 (83.5%) agreed that the term “heavy menstrual bleeding (HMB)” should replace the term “menorrhagia” for the symptom of excess menstrual bleeding.
- agreement on the principles, structure, and content of a “discussion” document for “Classification of causes of abnormal uterine bleeding.”
- Format and content of a proposed “Structured menstrual history” with widespread applicability.

## The Two Figo Aub Systems

In 2011, recognizing the international unmet need created by the impact of AUB, the FIGO MDWG published two systems (FIGO Systems 1 and 2) and a set of clinical recommendations in order to inform and aid clinicians and investigators in the design and interpretation of investigations into AUB in the reproductive years, as well as the provision of evidence-based clinical care (11). In 2012 FIGO endorsed the systems and, at the same time, “promoted” the MDWG to a standing committee called the “Committee on Menstrual Disorders,” or the “Menstrual Disorders Committee” known as the MDC. FIGO's Systems 1 and 2 are living entities designed to adapt to the evolving nature of menstrual norms and the classification process in light of ongoing debate and the assimilation of new knowledge from appropriately designed research. The most recent update was published in 2018 where the contributions from the FIGO MDC, as well as epidemiologists, gynecologists, and other experts from around the world between 2012 and 2017 were utilized. Where major change was considered, anonymous voting, in some instances using a modified RAND Delphi technique (described previously), was utilized (12).

### Terminology and Definitions (FIGO-AUB System 1)

So, what specifically is FIGO AUB System 1? To start with, System 1 describes non-gestational abnormal uterine bleeding (AUB) in the reproductive years as an overarching term for disturbances in one or more aspects of menstruation including the frequency, regularity, duration, and volume of menses including the presence of bleeding between periods and unanticipated bleeding associated with the use of medications such as gonadal steroids for contraception. The objective measurement of the volume of menstrual blood loss correlates poorly with presenting symptomatology and health seeking behavior. Consequently, FIGO has adopted the National Institute for Care Excellence definition of heavy menstrual bleeding (HMB) which, for clinical purposes, defines it as “excessive menstrual blood loss which interferes with the woman's physical, emotional, social and material quality of life, and which can occur alone, or in combination with other symptoms” (22).

In the original system published in 2007, FIGO introduced the concept of acute non-gestational AUB in the reproductive years, distinguishing it from chronic AUB. These definitions remain unchanged for 2018. Chronic non-gestational AUB in the reproductive years is defined as “bleeding from the uterine corpus that is abnormal in duration, volume, frequency, and/or regularity, and has been present for the majority of the preceding 6 months.” Acute AUB, on the other hand, is defined as “an episode of heavy bleeding that, in the opinion of the clinician, is of sufficient quantity to require immediate intervention to minimize or prevent further blood loss.”

When AUB occurs between well-defined cyclical episodes of menstrual bleeding, the symptom described as intermenstrual bleeding and may be further sub divided as:

- *Cyclic Midcycle IMB*—Small quantity of frank vaginal bleeding or discharge around midcycle. This may be physiological due to the nadir in circulating oestradiol levels that follow the oestradiol surge that initiates ovulation.
- *Cyclic Pre or Postmenstrual IMB*—Cyclical IMB that predictably occurs either early in the cycle (follicular phase) or late (luteal phase), and typically presents as very light vaginal bleeding for one or more days.
- *Acyclic IMB*—When the IMB is not cyclical or predictable.

**Figure 2:**
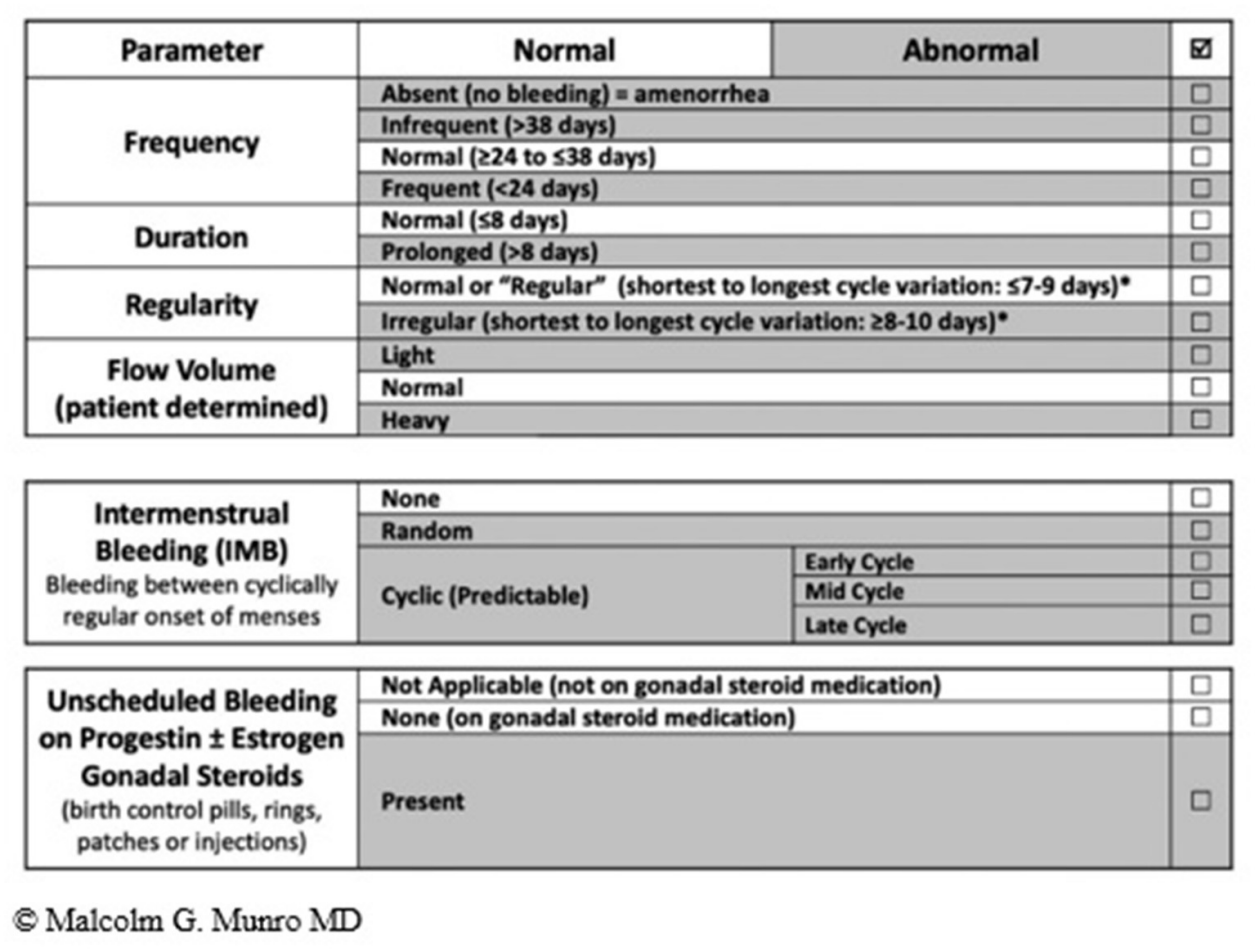
FIGO AUB System 1 Nomenclature and Definitions of AUB symptoms—The normal menstrual cycle is based on 4 parameters i.e., frequency, duration, regularity, and volume (subjectively determined by patient). The table shows normal values in unshaded areas and abnormalities in each of the parameters in shaded areas. The middle and lower panels are new (vs. 2011 paper); the middle panel is used to describe the presence or absence of IMB, whereas the lower panel is for the description of unscheduled bleeding while using gonadal steroid medication, most often progestogen or estrogen and progestogen-containing preparations.

The summary of the terminology recommended by the FIGO MDC is shown in Figure 2 (12).

### Classification of Causes of AUB in the Reproductive Years, the PALM-COEIN System (FIGO-AUB System 2)

**Figure 3:**
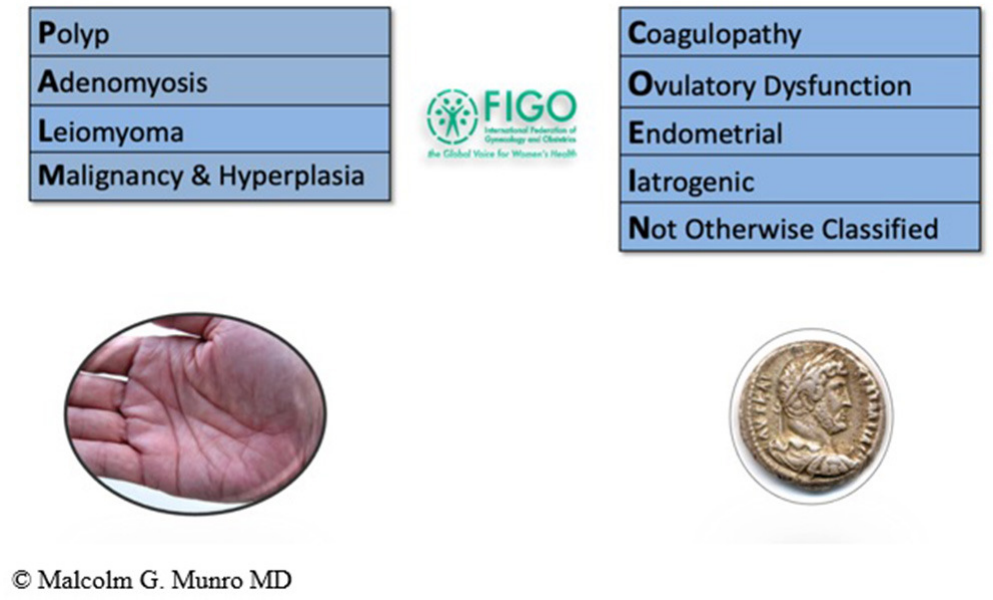
FIGO AUB System 2. PALM-COEIN system for classification of causes of AUB in the reproductive years. Adapted from Munro et al. (12).

System 2 describes the known potential causes or contributors to the symptoms categorized in System 1. There are nine main categories, arranged according to the acronym PALM-COEIN (pronounced “palm-koin”): Polyp; Adenomyosis; Leiomyoma; Malignancy and hyperplasia; Coagulopathy; Ovulatory dysfunction; Endometrial disorders; Iatrogenic; and Not otherwise classified. Since the original publication in 2011, category N has undergone a change from “not yet classified” to “not otherwise classified” recognizing that some entities may never have a specific classification category. The components of the PALM group are generally discrete (structural) entities that can be evaluated or measured visually using some combination of imaging techniques and histopathology; the COEI group comprises entities that are not defined by imaging or histopathology (non-structural). By its nature, the “Not otherwise classified” category includes a spectrum of potential entities that may or may not be measured or defined by histopathology or imaging techniques, but are not considered qualified for their own category or inclusion in an existing category (12) (see Figure 3).

The FIGO MDC is currently working on an international consensus for an imaging-based adenomyosis classification system designed to phenotype the disorder in a standardized fashion. However, for diagnosis the use of the transvaginal ultrasonography-based MUSA criteria have been defined (23).

#### Polyps (AUB-P)

Endometrial polyps are epithelial proliferations arising from endometrial stroma and glands (24). The reported prevalence of endometrial polyps ranges from 7.8 to 34.9%, depending on the definition of a polyp, the diagnostic method used, and the population studied (25–28). Exocervical polyps may be diagnosed by clinical examination, but those within the uterine cavity by one or a combination of ultrasonography, sonohysterography (US with simultaneous infusion of contrast into the endometrial cavity), hysteroscopy and histopathology. Blind endometrial sampling may identify polyps, however, have a low accuracy as compared to hysteroscopic directed biopsies (29, 30). Hysterosalpingography has a high sensitivity (98%), yet low specificity (35%) compared with hysteroscopic diagnosis (31). The gold standard for diagnosis of intrauterine polyps is hysteroscopy with a guided biopsy. Diagnostic hysteroscopy alone only has a reported sensitivity of 58–99%, specificity of 87–100%, positive predictive value (PPV) of 21–100%, and NPV of 66–99% when compared with hysteroscopy with guided biopsy as a diagnostic tool (32, 33).

#### Adenomyosis (AUB-A)

Adenomyosis is present when endometrial-like glands and stroma are identified in the myometrium, and is associated with hypertrophy and hyperplasia of the myometrium surrounding the ectopic endometrial tissue. The genesis of adenomyosis remains unclear, along with its association with AUB and infertility. Consequently, the appropriate diagnosis and management of adenomyosis remains poorly understood. Given the many uncertainties surrounding this condition, it has been recently described as an enigma (34).

Traditionally, the diagnosis of adenomyosis was made in retrospect, following histopathological assessment after hysterectomy for AUB. Defined sonographic criteria and magnetic resonance imaging (MRI) criteria for diagnosis of adenomyosis are described (23, 35). Despite this, the prevalence however remains unclear with a reported a 5–70% occurrence in histological diagnosis in hysterectomy specimens (36). Recent metanalyses have compared the accuracy of various imaging modalities in the non-invasive diagnosis of adenomyosis. Tellum et al. observed that pooled MRI, 2D-TVUS, and 3D-TVUS had a sensitivity of 78, 74, and 84% and a specificity of 88, 76, and 84% for diagnosing adenomyosis, respectively. 3D-TVUS could detect changes in the JZ, which was one of the more important diagnostic determinants (37). There was no statistically significant difference between the diagnostic quality of MRI and TVUS (35, 37).

Recent evidence illustrates that adenomyosis may also be present in a nearly a third of young (<30 years) nulliparous women with symptoms of HMB and /or dysmenorrhoea (38). Adenomyosis may also co-exist in up to 60% of women with severe forms of endometriosis when evaluated using MRI (39). Studies using ultrasound have also found a similar relationship between ovarian endometriosis and adenomyosis in young women (<30 years) (40). This emerging evidence dispels the previously held belief that adenomyosis is largely a disease of parous women in the 4 or 5th decade of their life, Newer modalities such as elastography (ultrasound mode) have made progress in reaching a diagnosis (41). There remains limited evidence to guide the management of women with adenomyosis, either medically or surgically.

#### Leiomyomas (AUB-L)

Leiomyomas (fibroid, myoma), are very common with the estimated cumulative incidence by age 50 is >80% for black women and nearly 70% for those who are white (42). Fibroids may be asymptomatic (incidentally diagnosed) or commonly contribute to AUB when submucous (43–45).

Uterine fibroids may be diagnosed by clinical examination, which may reveal an enlarged uterine or pelvic mass. The most common modality used in the diagnosis of uterine fibroids is ultrasound (US), which may be transabdominal (TA) or transvaginal (TV). Its low cost and accessibility often make it primary choice as a diagnostic modality. TV US is considered more sensitive than TA US for detection of small fibroids, for submucous fibroids and in obese patients (46, 47). The reproducibility, sensitivity, and specificity of US lacks consistency between different studies. Sensitivity and specificity ranged from 24–96 to 29–93%, respectively, in published literature (47). A recent meta-analyses observed that saline infusion sonography (SIS) has a pooled sensitivity in the detection of all intrauterine abnormalities (polyps, sub-mucous fibroids, adhesions) of 0.88 [95% confidence interval (CI): 0.85–0.90] and a pooled specificity of 0.94 (95% CI 0.93–0.96) and is comparable to hysteroscopy in this context (48). The sensitivity and specificity of SIS have been reported to be as high as 85–91 and 83–100%, respectively (47). The current NICE guidance recommends hysteroscopy as a first line investigation for AUB in women with suspected submucous fibroids vs. a TV US and thereby remains a gold standard in the diagnosis of suspected intrauterine pathology or where an endometrial biopsy is indicated (22). Magnetic resonance imaging (MRI) has the highest sensitivity and specificity (88–93, 66–91%), respectively, in the diagnosis of fibroids and differentiating fibroids from focal adenomyosis when compared to other discussed modalities. It has excellent reproducibility vs. US, SIS and hysteroscopy and can identify unusual fibroids e.g., parasitic fibroids, and the extent of fibroid degeneration. Despite these advantages, the routine use is precluded by cost and accessibility (47).

**Figure 4:**
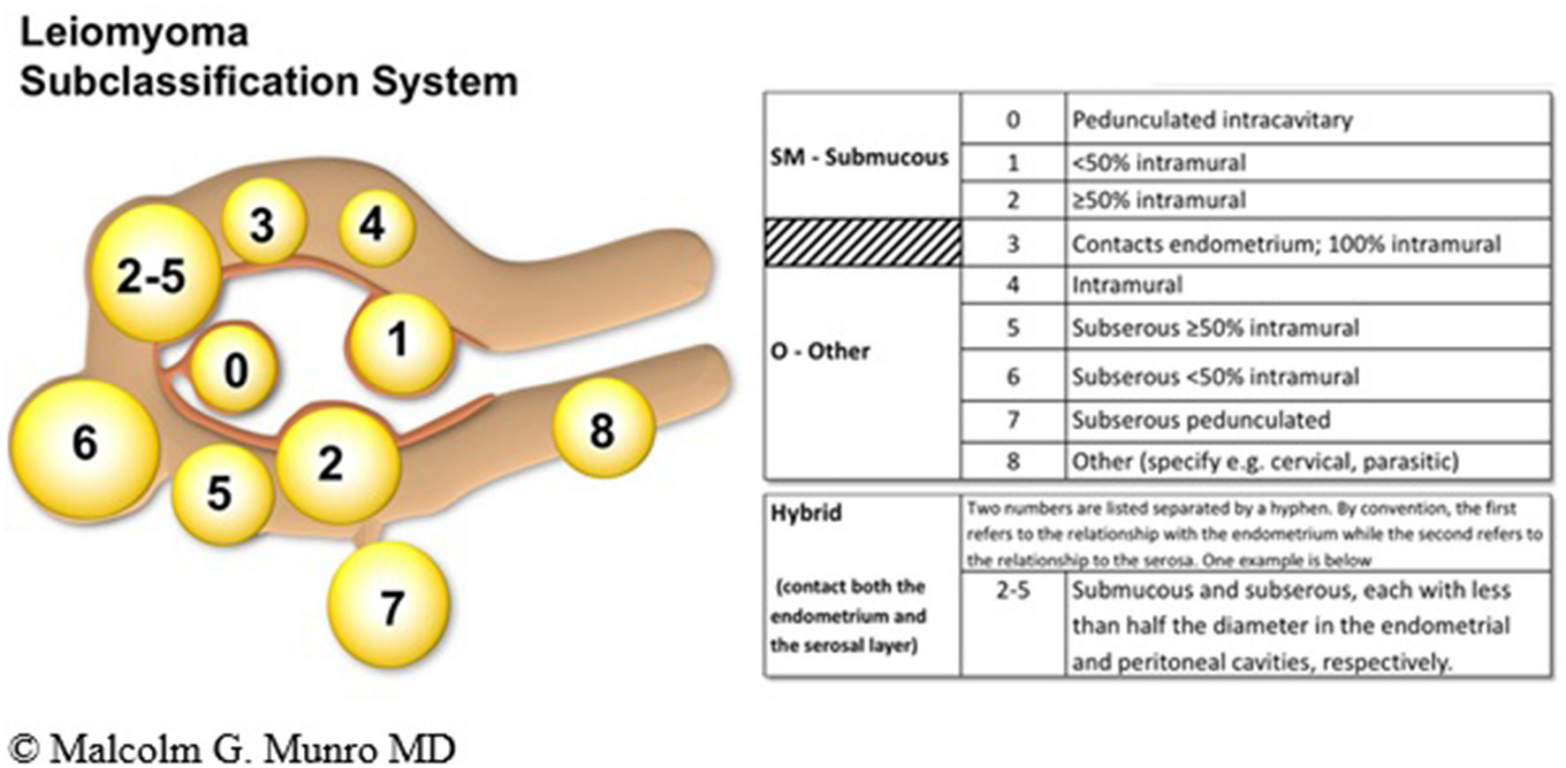
FIGO leiomyoma sub-classification system. Adapted from Munro et al. (12).

The FIGO PALM-COEIN leiomyoma system is extensive and to date, the only sub-classification to be ratified by the FIGO. The system includes primary, secondary, and tertiary classification of leiomyomas with the first level being presence or absence, the second submucous or “other” and the third a categorization that includes the submucous group according to the original Wamsteker et al. system (49). The FIGO system adds additional categorisations for submucous, intramural, subserosal, and transmural lesions. Intracavitary lesions are attached to the endometrium by a narrow stalk ( ≤ 10% or the mean of three diameters of the leiomyoma) and are classified as Type 0, whereas Types 1 and 2 require a portion of the lesion to be intramural—with Type 1 being <50% of the mean diameter and Type 2 at least 50%. Type 3 lesions are intramural but also abut the endometrium. Although they can be diagnosed with imaging techniques such as sonohysterography and MRI, Type 3 lesions are formally distinguished from Type 2 with hysteroscopy using the lowest possible intrauterine pressure necessary to allow visualization. Type 4 lesions are intramural leiomyomas that are entirely within the myometrium, without extension to the endometrium or to the serosa. Subserous (Types 5, 6, and 7) leiomyomas represent the mirror image of the submucous leiomyomas—with Type 5 being at least 50% intramural, Type 6 being <50% intramural, and Type 7 being attached to the serosa by a stalk that is also ≤ 10% or the mean of three diameters of the leiomyoma. Classification of lesions that are transmural are categorized by their relationship to both the endometrial and the serosal surfaces. The endometrial relationship is noted first, with the serosal relationship second (e.g., Type 2–5). An additional category, Type 8, is reserved for leiomyomas that do not relate to the myometrium at all, and would include cervical lesions (demonstrated), those that exist in the round or broad ligaments without direct attachment to the uterus, and other so-called “parasitic” lesions (12) (see Figure 4).

Location seems to be a more important factor than size in determining bleeding symptoms. Submucous myomas, those in or partially intruding into the endometrial cavity, are most likely to cause heavy menstrual bleeding. The reason why these tumors cause disproportionate bleeding is not clear (44).

#### Malignancy and Hyperplasia (AUB-M)

FIGO System 2, the PALM-COEIN system, aims to complement pre-existing classification systems by the World Health Organization (WHO) and FIGO for atypical endometrial hyperplasia (also known as endometrial intraepithelial neoplasia, or EIN) and gynecological malignancies, in particular endometrial cancer (50, 51). There are several risk factors for EIN and endometrial cancer in premenopausal women that include obesity, a family history, and chronic anovulation from a spectrum of causes that typically manifest with irregular menstrual bleeding (AUB-O). These have been defined by the RCOG (52) and reflect the increasing incidence relating to the increased prevalence of obesity in many populations (53). WHO first proposed a classification system for endometrial hyperplasia in 1994 (54), which was subsequently revised in 2004 (55).

The current NICE guidance recommends that women presenting with AUB, where an endometrial biopsy is deemed necessary, this should be done in the context of outpatient hysteroscopy, rather than blind sampling (22). The high accuracy, sensitivity, and specificity of hysteroscopy in assessing intrauterine pathology are well-studied (56, 57).

Cervical cancer may present as persistent IMB or post-coital bleeding.

Leiomyosarcoma (LMS) is an aggressive uterine tumor (sarcoma) and may present with AUB often associated with a rapid increase in fibroid size. The incidence of uterine sarcoma is a topic of current interest and good quality data are required, specifically given the high utilization of power morcellation of fibroids during minimal access surgery. These tumors are aggressive, have a poor prognosis and a high recurrence rate following treatment, with intraperitoneal dissemination having potentially disastrous consequences due to seeding of malignant cells. Age and peri-menopausal status are important considerations. Recent data highlights an increased incidence of expected uterine sarcomas for women undergoing hysterectomy for benign indications, including fibroids (58–60). This risk increases with age and the risk is higher in women >45 years (58, 60). As the evidence base concerning risk of LMS in women with uterine fibroids builds important information will be available to clinicians to inform management discussions. This is an important finding as it provides important insights in clinical practice in guiding management in women with fibroids i.e., an informed discussion of the potential risks of a conservative approach (fibroid surveillance) in older women. Symptomatic postmenopausal women with uterine fibroids represent a particularly high-risk group (61) and may need more definitive treatment.

At the present time, there remains no laboratory test, for example, a tumor maker or an imaging study (ultrasound, MRI, CT scan) that can reliably diagnose uterine LMS preoperatively (62).

Recent evidence emphasizes the importance of performing endometrial sampling in women with AUB with suspected benign disease. Although the likelihood of diagnosing uterine sarcomas is low, a liberal approach to endometrial sampling may reduce the risk of unexpected non-benign histology in women undergoing hysterectomy (60). Furthermore, younger women who are obese are also at a risk of endometrial cancer and as such should be considered for endometrial sampling (63).

#### Coagulopathy (AUB-C)

Underlying bleeding disorders are reported to affect 12–14% of the women presenting with the symptom of HMB, most commonly von Willebrand disease (64). While it is generally perceived that these diagnoses are made in adolescence, around menarche, when subtle abnormalities exist the first presentation of AUB-C may occur in adult life. A simple set of screening questions may allow identification of women at high risk, such that an appropriate laboratory testing can be performed, with or without onward referral to a hematologist. The system presented is 90% sensitive for the presence of a coagulopathy (14, 65) (see Table 3).

**Table 3:** Screening for hematological abnormalities in women with AUB.

**Structured history—positive screen if**
a. Excessive menstrual bleeding since menarche, or
b. History of one of the following—postpartum hemorrhage, surgery-related bleeding, or bleeding associated with dental work, or
c. History of two or more of the following—bruising >5 cm once or twice/month, epistaxis once or twice/month, frequent gum bleeding, family history of bleeding symptoms

*Adapted from Kouides et al. (14)*.

Based on the screening results a secondary evaluation may need to be undertaken in consultation with a hematologist as summarized in Table 4. A primary full blood count should be undertaken in all women presenting with AUB/HMB and a normal platelet count should be established prior to the secondary evaluation below. The evaluation of thrombocytopenia's is beyond the scope of this chapter.

**Table 4:** Screening for hematological abnormalities in women with AUB.

1	a. PT and APTT (if APTT prolonged, do mixing assay for inhibitor or factor deficiency)
	b. VWF antigen
	c. ristocetin cofactor d. Factor VIII
	e. ABO type
	f. Ivy bleeding time and/or PFA-100 closure time
	Nonhematologic testing: Consider TSH, especially if VWF levels reduced, and baseline iron profile if anemic prior to intervention
2	If #1 is normal, then consider platelet aggregation and release studies
	If #2 is normal, then consider specific factor levels (e.g., FXI, FXIII), and euglobuin clot lysis and other measures of fibrinolysis (α_2_-antiplasmin level, plasminogen activator inhibitor level)
	For females without positive screen as noted in structured history above, but who are considering major surgical intervention, consider secondary evaluation. This because up to 8% of women without a positive screen (Table 3) will have underlying VWD (65)

*Adapted from Kouides et al. (14)*.

#### Ovulatory Disorders (AUB-O)

Ovulatory disorders comprise a spectrum of disturbance in normal ovulatory function ranging from irregular or infrequent ovulation to anovulation. By its nature anovulation results in exposure of the endometrium to various levels of unopposed estrogen, which, absent progesterone, typically result in a persistent proliferative state and a consequent increase in the incidence of endometrial hyperplasia. Women with ovulatory disorders may be amenorrheic (a term retained by FIGO) or can manifest with infrequent and/or prolonged cycles and bleeding of a variety of durations and volumes, either related to spontaneous endometrial sloughing or to periodic ovulation and progesterone withdrawal. AUB-O is common in the early years following menarche and again during the perimenopausal transition due to changes in the hypothalamic-pituitary-ovarian axis, typically evolving to cyclical bleeding in the adolescent and, with menopause, the onset of amenorrhea. Ovulatory disorders may also be associated with or caused by other conditions such as hypothyroidism, hyperprolactinemia, extremes of weight (including sudden changes in weight), mental stress, and excessive exercise. The diagnosis of ovulatory disorders is largely based on a detailed menstrual history that is descripted in FIGO System 1. The use of serum progesterone measured in the presumed luteal phase, or the results of endometrial sampling may have occasional utility but can also be misleading since they reflect only a single cycle.

The recent FIGO classification systems update (2018) recommends that therapies interfering with the H-P-O axis and associated with AUB, now be placed in the “AUB-I” category.

#### Endometrial (AUB-E)

AUB that occurs with regular menstrual cycles in the absence of a bleeding disorder and unrelated to structural abnormalities is likely to represent a primary endometrial disorder. The exact etiology remains poorly understood, although defective local haemostasis may contribute (66–68). It is important to understand that structural anomalies such as uterine leiomyomas NOT in contact with the endometrium are unlikely to contribute to AUB, and in such instances, AUB-E or AUB-O should be considered depending on the characteristics of the menstrual cycle. There are no validated tests currently available for clinical use to diagnose AUB-E, which is a primary disorder of endometrial haemostasis. It is a diagnosis when no other explanation is found following clinical assessment (history, physical examination) conduct of appropriate blood tests and uterine imaging.

#### Iatrogenic (AUB-I)

AUB-I occurs secondary to the use of several medications or to the use of intrauterine systems, typically designed for contraception, but also those used primarily for the treatment of selected causes of AUB. They may be allocated to one of 5 major categories.

- Exogenous gonadal steroids, including levonorgestrel-releasing intrauterine systems (LNG-IUS), long-acting progestin preparations e.g., etonogestrel implants, gonadotrophin releasing hormone modulators including agonists and antagonists. These drugs alter the prevailing endocrine environment and often contribute to unscheduled or breakthrough bleeding (69). Up to 1 in 5 women using progestin only contraception may develop AUB-I (70). Hormonal polytherapy may also be contributory.
- Pharmaceutical agents that alter drug bioavailability by modifying hepatic enzyme metabolism. Examples include anti-epileptic or anti-tuberculous drugs, which may alter the circulating level of gonadal steroids.
- Anticoagulants such as warfarin, unfractionated heparin, low molecular weight heparin with impaired formation of an adequate “plug” or clot within the vascular lumen.
- Agents that impact dopamine physiology. These include tricyclic antidepressants (e.g., amitriptyline and nortriptyline) and phenothiazines that can result in hyperprolactinemia with subsequent ovulatory dysfunction.
- Inert intrauterine systems or that contain copper or alloys that are designed for contraception.

#### Not Otherwise Classified (AUB-N)

On occasion AUB may be associated with rare or uncommon conditions or those for which there is an unclear association with symptoms. Worldwide, the incidence of cesarean delivery (CD) is rising substantially, and it has been recognized that there exists in many a defect at the incision site on the uterus that has been called variously a niche, an isthmocele or simply a cesarean scar defect (CSD). These defects at the site of CD may contribute to AUB and FIGO is currently undertaking a systematic review to study this relationship as a prelude to considerations of how and if this putative mechanism should be included in FIGO System 2 (71, 72). Uterine arteriovenous malformations may also be responsible for acute uterine bleeding but are not known to contribute to chronic AUB in the reproductive years (73, 74).

## Concluding Thoughts

The two FIGO classification systems are designed to define the nomenclature used to describe menstrual symptoms (System 1) and with System 2, to categorize the potential underlying causes or contributors to the spectrum of symptoms described in System 1. Indeed, it is important to understand that FIGO System 1 should be considered to be a mandatory gateway to the application of System 2, since many diagnoses require clear description of the menstrual symptoms experienced by the woman. This approach is clinically important in instituting the most appropriate approach to investigation and to the identification of a menu of treatment options that can be tailored to the individual patient considering her current clinical situation, future desires regarding fertility, and to her cultural and religious norms. In addition to helping the practicing healthcare professional manage patients with AUB, including the coordination of care, the two systems are also excellent tools for teaching and training due to the simplified expression of the concept of AUB. Since their introduction, the FIGO systems have received worldwide acceptance; at the time of writing this article there are approximately 2,444 citations in the literature, 1,480 of the two IJGO papers (11, 12). The systems are designed to be flexible with further classifications and subclassifications proposed in the future, thus allowing clinicians to refine and provide optimum care to patients, and bench, epidemiological and clinical investigators a structure within which to design and interpret AUB-related research.

## Author Contributions

All authors listed have made a substantial, direct, and intellectual contribution to the work and approved it for publication.

## Funding

HC receives support from Medical Research Council Centre for Reproductive Health (MRC CRH) Grant MR/N022556/1.

## Conflict of Interest

HC has received clinical research support for laboratory consumables and staff from Bayer AG (paid to Institution) and provides consultancy advice (no personal remuneration) for Bayer AG, PregLem SA, Gedeon Richter, Vifor Pharma UK Ltd, AbbVie Inc, and Myovant Sciences GmbH. HC receives royalties from UpToDate for article on abnormal uterine bleeding. RC has been supported as a clinical research fellow by Bayer AG. MM has received research support from AbbVie Inc and Pharmacosmos, and for consulting services from AbbVie Inc, Daiichi-Sankyo, Hologic Inc, Myovant Sciences, Pharmacosmos, and Vifor Pharma.

## Publisher's Note

All claims expressed in this article are solely those of the authors and do not necessarily represent those of their affiliated organizations, or those of the publisher, the editors and the reviewers. Any product that may be evaluated in this article, or claim that may be made by its manufacturer, is not guaranteed or endorsed by the publisher.
